# Molecular Characterization of Lumpy Skin Disease Virus Isolates from Outbreak Cases in Cattle from Sawena District of Bale Zone, Oromia, Ethiopia

**DOI:** 10.1155/2021/8862180

**Published:** 2021-02-04

**Authors:** Shubisa Abera Leliso, Fufa Dawo Bari, Tesfaye Rufael Chibssa

**Affiliations:** ^1^National Animal Health Diagnostic and Investigation Center (Nahdic), Sebeta, Ethiopia; ^2^Addis Ababa University College of Veterinary Medicine and Agriculture Department of Microbiology, Immunology and Veterinary Public Health, Bishoftu, Ethiopia

## Abstract

Lumpy skin disease (LSD) is a viral disease caused by LSD virus and is one of the most economically significant transboundary and emerging diseases of cattle. LSD causes considerable economic losses due to emaciation, damage to hides, infertility, and loss of milk production. In Ethiopia, the disease is distributed almost in all regions and is regarded as one of the most economically important livestock diseases in the country. An outbreak investigation of the disease was monitored from October 2016 to April 2017 in southern pastoral areas of Bale Zone, Oromia, Ethiopia. In December 2016, LSD outbreak occurred in Sawena district of Bale Zone, from which necessary biopsy samples were collected from actively infected animals for the purpose of virus isolation, and characterization using different molecular techniques at National Animal Health and Diagnostic Investigation Center (NAHDIC) of Sebeta, Ethiopia. In addition, clinical examination of infected and in-contact animals was carried out together with a questionnaire survey. Based on the clinical manifestations, LSD was recorded in 18% (94/522) of examined cattle, whereas biopsy samples from 20 clinically positive animals were collected for further laboratory process. The morbidity rate was higher in animals less than two years 28.97% (31/107) than other ages and showed a statistically significant difference with *P* < 0.05. Female animals showed higher morbidity rate of 20.59% (76/369) than male animals (11.76%) (18/153) with a significant difference at *P* ≤ 0.003. Mortality rate and case fatality were also significantly higher in young animals than other age groups. Viruses were isolated from both skin biopsies and nasal swabs on Vero cell line. From both skin biopsies and nasal swabs, the virus DNA was identified by amplifying the 172 bp DNA fragment using real-time and conventional PCR. Providing adequate diagnostic facilities, establishing strategic policies for effective control and eradication and awareness creations for communities for early identification or reporting were recommendations made to minimize economic losses of the disease.

## 1. Introduction

In the Greater Horn of Africa (GHA), pastoralists occupy large parts of arid and semiarid lands of Ethiopia, Kenya, Somali, Djibouti, Eritrea, Sudan, Uganda, and Tanzania [1]. Together with agropastoralists, they comprise significant proportions of national populations in each of these countries [2]. Ethiopia has the largest livestock population in Africa, possessing more than 57.8 million cattle, 56.6 million small ruminants, 1.2 million camels, 10.4 million equines, and 60.5 million chickens.

This livestock sector has contributed considerable portion to the economy of the country and is still promising to rally round the economic development of the country. Livestock production remains crucial and represents a major asset among resource-poor small holder farmers by providing milk, meat, skin, and manure and traction force [3]. The contribution of livestock to the national economy particularly with regard to foreign currency earnings is through exploration of live animal, meat, and skin and hides [4].

In the highlands, livestock are kept under settled or transhumant systems utilizing common pastures, many of which have a high clover content and crop residues. Such livestock include some 9.3 million oxen providing draught power for the mixed farming system that prevails. In the arid and semiarid extensive grazing areas of the eastern, western, and southern lowlands, cattle, sheep, goats, and camels are managed in migratory pastoral production systems [5].

The Bale pastoralists' livelihoods depend predominantly on livestock and their products. They practice a transhumance nomadic system, which had been their traditional and primary survival strategy. However, physical infrastructure is poorly developed in areas where pastoralists live. Poor health and productivity of animal due to disease has considerably become the major stumbling block to the potential of livestock industry [2].

LSD is one of the most economically significant transboundary, emerging viral diseases. It is currently endemic in most Africa countries and expanded to the Middle East region [6]. It is a disease with a high morbidity and low mortality rate and affects cattle of all ages and breeds. It causes significant economic problems as a result of reduced milk production, beef loss and draft animals, abortion, infertility, loss of condition, and damage to the hide [7]. It becomes an important threat to livestock and dairy industry in the Middle East and Africa [8].

The disease is now the problem of almost all the regions and agroecological zones of Ethiopia. A major outbreak of LSD has occurred in different regions of Ethiopia like Amhara and W/Oromia regions in 2000/2001, Oromia and Southern nations' nationalities and people (SNNP) regions in 2003/2004, and Tigray, Amhara, and Benishangul regions in 2006/2007 [9]. LSD is an OIE-listed disease because of considerable financial losses and in Ethiopia due to the endemic nature of LSD; the country is facing serious difficulties in exporting live cattle and their products. In addition, this situation contributes a negative impact on the national economic growth through the loss of meat and milk production and poor quality of skin and hides [10].

Hence, it is important to generate information on isolation of LSD from outbreak reported and its molecular characterization. Therefore, the objectives of this study are to investigate the occurrence of LSD outbreak in pastoral areas and to isolate the infectious LSDV from nasal swab and skin lesion samples in Vero cell cultures in the study area.

## 2. Materials and Methods

### 2.1. Study Area

The outbreak investigation was conducted from November 2016 to April 2017 in Bale Administrative Zone of Oromia Regional State, which is located 430 km southeast from the capital city, Addis Ababa. The town of the Robe Zone is geographically found at 7°7′N 40°0′E with an elevation of 2,492 meters above sea level. Bale Zone of Oromia Regional State was purposively selected for the study based on LSD outbreak report to Yabello Regional Veterinary Laboratory and Zonal pastoral office.

**Figure 1:**
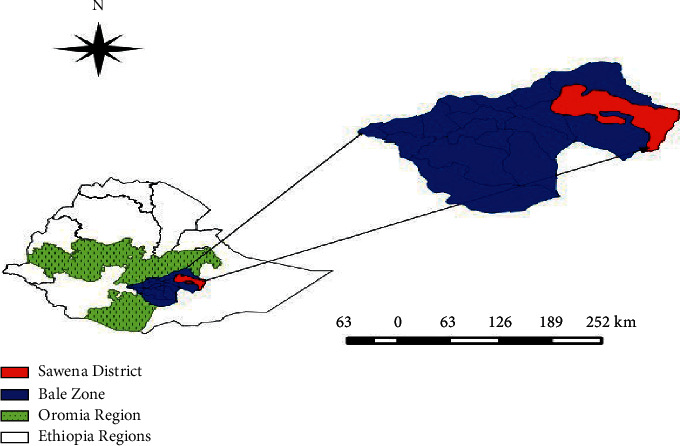
Map of the study area.

The altitude of the study area ranges from 850 to 2800 m.a.s.l, where the lowland area predominates with a narrow strip of high land area in the northern part of Sawena district (Figure 1). The area experiences a bimodal rainfall occurring from September to November and March to June. An average annual temperature of 20–25 °C and rainfall of 200 mm are recorded in Sawena district. Surface water is a serious problem in Sawena district, where only seasonal streams, ephemeral ponds, and shallow temporary wells are sources of water in the rainy season and these usually dry out after a few days during the dry season. Sawena district has a pastoral vocation with livestock rearing being the dominant economic activity of the district.

### 2.2. Study Animals

The active outbreak investigation was conducted in extensive management system in local cattle. Cattle that showed clinical signs of pox like skin lesion were targeted for this study. All age and sex groups which are reared under two different production and management systems (small number of animals especially milking cows and calves that are kept around the home and the majority of animals which are driven long distances in search of good pasture and surface water) were involved in the outbreak investigation.

### 2.3. Study Design

The study areas were selected purposively based on the reports of LSD outbreaks to Yabello Regional Veterinary Laboratory. Active outbreaks were assessed together with veterinary professionals who are working in the regional veterinary laboratories, zonal veterinary office, and district veterinary clinics. Clinical and epidemiological data were recorded and samples were collected for virus isolation and identification that were conducted at Animal Health and Diagnostic Investigation Center (NAHDIC) at Sebeta, Ethiopia.

#### 2.3.1. Questionnaire Survey and Epidemiological Data Collection

Additionally, semistructured questionnaires were used to interview 20 pastoralists on LSD occurrence and its associated impacts. The cattle owners expressed their views and shared their practical knowledge about the prevailing situations regarding LSD using their native language (Afan Oromo); among the questions were awareness about clinical signs of LSD, age affected, sex affected, seasons of LSD outbreak, livestock movement, milk reduction, abortion in pregnant cows, presence of death due to the disease, status of vaccination, and water sources for the livestock. The pastoralist called the local name of the diseases “Itesa.” Relevant data were gathered by observing clinically sick animals and interviewing cattle owners and animal health workers working at the field. Information was carefully recorded in a designed format.

### 2.4. Sample Size and Sampling Technique

During the study period, the total number of animals examined was 522. A field investigation was conducted purposively at the specific site of the outbreak within Bale Zone. Cattle with clear signs and symptoms and suspected to be diseased with LSD were selected to be sampled. Skin nodules from cattle which were with severe clinical signs of the disease were taken aseptically by washing and cleaning the area and removing the hairs with the help of sterile scalpel blade. Nasal swabs were collected with sterile swabs from infected animals.

### 2.5. Sample Collection and Transportation

According to the procedures of OIE [11], samples for virus isolation and molecular characterization were collected from clinically sick animals. From a total of twenty samples, seven skin nodules and thirteen nasal swabs were collected. The representative samples were aseptically collected from infected cattle with typical developed severe clinical signs of the disease.

Skin nodules were taken aseptically by washing and cleaning the area and removing the hairs with the help of sterile scalpel blade and nasal swabs were taken aseptically by cleaning the external part of noses. Tissue samples were placed in the sterilized universal bottle containing virus transport medium and nasal swabs were placed in the sterilized cryovial tubes containing virus transport medium kept at −20°C until transported to National Animal Health Diagnostic and Investigation Center, Sebeta, Ethiopia.

### 2.6. Laboratory Techniques

#### 2.6.1. Sample Processing

The skin biopsy samples were thawed at room temperature and washed three times in sterile phosphate-buffered saline (PBS, pH 7.2). Approximately 1 g washed tissue sample was mixed with 9 ml sterile PBS and grounded using a sterile mortar and pestle. The tissue suspension was centrifuged at 4000 rpm for 15 min. And the supernatant was filtered through a membrane of pore size 0.45 *μ*m [12].

#### 2.6.2. Virus Isolation

Approximately 1 ml filtered supernatant was inoculated onto a monolayer of Vero cells in 25 cm^2^ tissue culture flasks, incubated at 37°C for an hour for adsorption, and then 9 ml Glasgow minimum essential medium (GMEM, Sigma-Aldrich), containing 0.1% gentamicin and 2% fetal calf serum (Sigma-Aldrich), was added. The inoculated flasks were incubated at 37°C in a humidified incubator with 5% CO_2_. Cells were monitored daily for 14 days, using an inverted microscope, for evidence of virus-induced cytopathic effects (CPEs); finally, cells were frozen at –80°C [9, 12].

#### 2.6.3. DNA Extraction

DNA was extracted by Qiagen kit, according to the manufacturer's instructions. The tissue sample was cut into pieces and grinded with sterile sand by adding PBS buffer and after centrifugation at 2000 rpm for 2 minutes, the supernatant was collected into new microcentrifuge tubes; after that, 200 *μ*l of it was taken and 20 *μ*l of proteinase K was added, mixed by vortexing, incubated at 56°C for 10 minutes, and it was briefly centrifuged. Then, 200 *μ*l ethanol (96–100%) was added and mixed thoroughly for 5 seconds by vortex mixer and briefly centrifuged. This mixture was applied to the QIAamp mini spin column and centrifuged at 6000 xg (8000 rpm) for 1 minute. The spin column was transferred into 2 ml collection tube and 500 *μ*l buffer AW1 was added and then it was centrifuged at 8000 rpm for 1 minute; after that, 500 *μ*l buffer AW2 was added and centrifuged at a full speed of 14000 rpm for 3 minutes after discarding the filtrate. 200 *μ*l of buffer AE was added and incubated at room temperature for 1 minute and centrifugation continued at 8000 rpm for 1 min. This step was repeated to get the final extract.

#### 2.6.4. Polymerase Chain Reaction

Real-time polymerase chain reaction (RT-PCR) assay was used to detect the virus with *Capripoxvirus*-specific primers used.

Forward: 5″-GGTGTAGTACGTATAAGATTATCGTATAGAAACAAGCCTTTA-3″; reverse: 5″-AATTTCT-TTCTCTGTTCCATTTG-3″

DNA was amplified in a final volume of 20 *μ*l containing the following: 10 *μ*l of eva green super mix, containing 2 *μ*l of forward and reverse primers (4 *μ*l), 4 *μ*l of RNase-free water, and 2 *μ*l of template DNA. The following amplification program was applied: initial denaturation at 95°C for 3 minutes, followed by 45 cycles at 95°C for 15 seconds, 58°C for 80 seconds, and last cycles of 95°C for 1 minute, 40°C for 1 minute, and 40–85°C for 5–10 seconds for melting curve analysis.

After amplification of the DNA template, the positive samples were noted by amplification fluorescence curves, melting curves (at 73°C), and cycle threshold (Ct) values from the assay which were used to describe the positive samples: Ct value higher than 40 was indicated as negative suggesting absence of the virus from the tissue specimens and nasal swabs [10].

#### 2.6.5. Agarose Gel Electrophoresis

The amplified DNA from extracted specimens was analyzed by agarose gel electrophoresis as described by [13] with some modification to confirm the presence of DNA. Amplified products were analyzed using a Gene Ruler^TM^ 100 bp DNA ladder (Fermentas, Germany) as a molecular marker on 2% agarose gels prepared in Tris/acetate/EDTA (TAE) buffer and 10 mg/ml ETDM-bromide strain, and then 20 *µ*l of PCR product was mixed with 4 *µ*l loading buffer and loaded to wells in gel and run at 100 volts for about 60 minutes in parallel with DNA molecular weight marker in the electrophoresis apparatus until the DNA samples have migrated a sufficient distance through the gel. DNA bands were visualized using an UV transilluminator at a wavelength of 590 nm, and positive results were confirmed according to the size of the bands formed on agarose gel. The PCR results were considered positive for LSDV and GTPV DNA when a 172 bp band was observed and for SPPV the band size is 151 bp [14].

### 2.7. Data Management and Analysis

The collected data were coded, entered, and stored into Microsoft Excel spreadsheet 2010. The data were thoroughly screened and properly coded before subjecting to statistical analysis. The data were imported from Microsoft Excel and analyzed using Statistical Package for Social Sciences (SPSS) software version 20. The morbidity and mortality were estimated in accordance with sex and different age categories.

Confidence interval was also used to describe morbidity and mortality across different variables. Chi-square (*X*^2^) was employed to test the presence of association among different categorized variables. In all the analyses, confidence levels at 95% were calculated, and a *P* ≤ 0.05 was used for statistical significance level [15]. Descriptive statistics was also used to quantify the results on awareness of community on importance of the disease, age and sex of the affected and dead cattle during the outbreaks.

### 2.8. Ethical Approval

Sampling from animals was carried out according to the experimental practice and standard approved by the Animal Welfare and Research Ethics Committee at Addis Ababa University College of Veterinary Medicine and Agriculture, Bishoftu Campus, which is in accordance with the International Guidelines for Animal Welfare, with verification number VM/ERC/25/06/09/2017.

## 3. Results

### 3.1. Observed Clinical Signs

**Figure 2:**
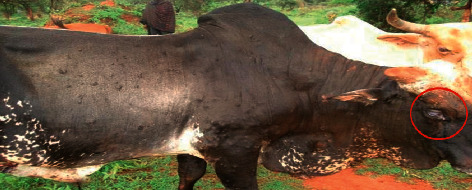
An inflammation and whiteness of the eye of cattle infected with LSD. Encircled area shows lacrimation probably because of secondary bacterial complication.

**Figure 3:**
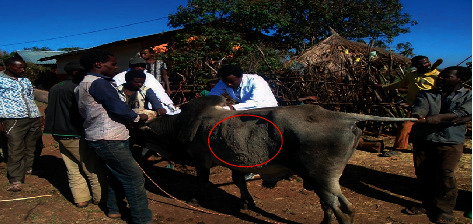
Characteristic signs of LSD with generalized circumscribed skin nodules covering the entire body. Encircled area shows developed circumscribed nodules on the skin. Pictures were taken during field investigation.

Out of 522 cattle examined, clinical manifestation relevant to LSD was recorded in 18% (94/522) of cattle. The common clinical signs observed in cattle affected by LSD were fever, development of different sizes of circumscribed nodules on the skin, necrotic nodules, deep scab formation, swelling of dewlap, and enlargement of superficial lymph nodes. Lacrimation (Figure 2), dewlap, and superficial lymph nodules enlargement were very prominent. Burst necrotic wounds were often complicated with secondary infection (Figure 3).

According to the result of study where outbreak of LSD was investigated, there were 18% morbidity, 1.34% mortality, and 7.44 case fatality rates observed in study area. In this study, a slightly higher morbidity rate was observed in Arda Galma kebele, but the mortality and case fatality are relatively lower (Table 1). The clinical cases of LSD were significantly higher in younger cattle (*P* ≤ 0.001) and in male animals (*P* ≤ 0.003) as compared with the other age and sex categories, respectively (Table 2).

The mortality observed among the different age groups was significant (*P* < 0.05) which was higher in young animals less than 2 years of age (Table 3).

### 3.2. Molecular Characterization

#### 3.2.1. Virus Isolation

**Figure 4:**
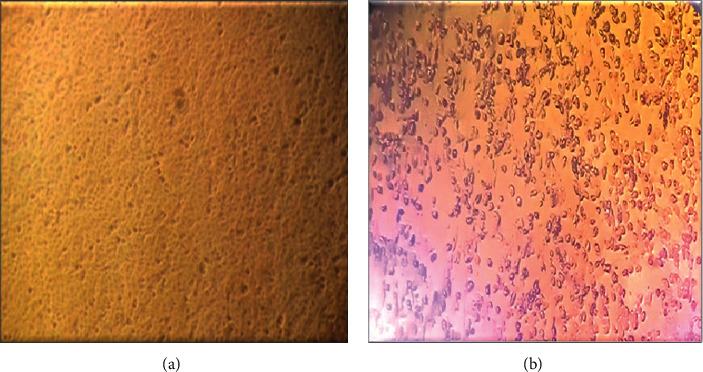
LSD growth on Vero cell culture. (a) Uninoculated Vero monolayer cell after 24 hours. (b) Infected Vero cells showing typical CPE at 11^th^ day.

Out of 20 samples, 7 skin biopsy samples and 13 nasal swabs were inoculated in Vero culture and the virus was passaged three times. Although it was not be able to isolate all samples, the characteristic LSDV CPE was perceived in most infected cell cultures (Figure 4). The CPEs were characterized by rounding of single cells, aggregation of dead cells, and destruction of monolayers.

#### 3.2.2. Polymerase Chain Reaction

**Figure 5:**
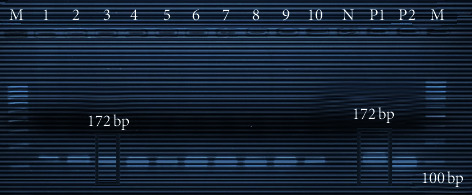
Classical PCR gel picture from skin nodule and nasal swabs of LSDV infected cattle. Lane M: DNA ladder (100bp, Fermentas); lanes 1–10 represent positive samples from Sawena district of Bale; N: negative control without template; P1: positive control for LSD; P2: positive control for SPP.

The extracted DNA of 20 specimens was amplified using *Capripoxvirus*-specific primers. The amplicon size of PCR product had a molecular weight of 172 bp (Figure 5), which is the expected amplicon size for the LSDV genomic region targeted. The resulting PCR products of LSDV uniformly aligned online suggesting they have the sample amplicons size.

#### 3.2.3. Conventional PCR

Conventional PCR was run targeting the RPO30 gene of the collected samples. Amplicons were analyzed by 3% agarose gel electrophoresis. The specific primers set amplified a DNA fragment of 172 bp equal to the expected amplification product size from LSDV. The result showed that the reference strain of the LSDV and the local isolate from skin nodules and nasal swabs had the same size of RPO30 gene fragment 172 bp.

#### 3.2.4. Real-Time PCR

**Figure 6:**
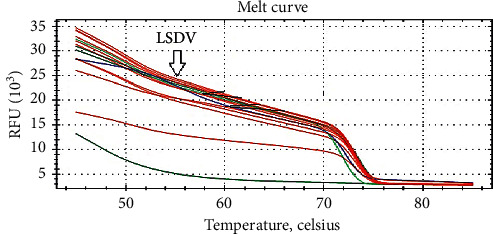
Melting curve analysis of field isolates of LSDV.

Melting curves are generated from the DNA of skin biopsy and nasal swabs sample with respect to known LSDV controls (Figure 6). As shown in the plots, the same melting profile as that of the LSDV reference strain, the amplicons 73.0°C, and the snapback 51.0°C was obtained for all screened isolates.

### 3.3. Questionnaire Survey

**Figure 7:**
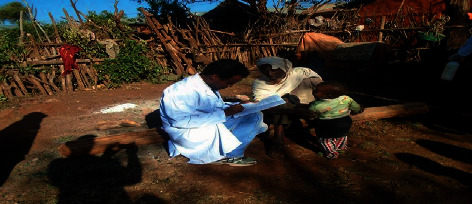
Picture taken during questionnaire survey in the study areas.

According to the pastoralist community, the local name for LSD was mentioned as “Itesa.” About 17 of the 20 respondents said that the LSD outbreak reoccurs in the frequency of three years, while 3 individuals said the disease occurs at an interval of two years. They also reported that the incidence of the disease increased during the rainy season (Figure 7). Concerning the possible source of infection, the pastoralist responded as the source could be different in that contact at communal points (like marketing, watering, and grazing) accounted for 45% (9/20), introduction of sick animals to the herd accounted for 30% (6/20), while the other 25% (5/20) pastoralists did not know of any source.

The survey enabled an estimation of the direct economic losses resulting from animals dying from LSD. Production losses were estimated from the weighted average price of each animal that died. An average cost of a single ox dying from this disease is 7,000 Ethiopian birr. For the one active outbreak investigated in two villages, the total economic losses from the deaths of 7 animals were 50,000 ETB (US$2174). The estimated total expense incurred for the treatment of LSD was also assessed. An average of 21 birr/animal was incurred for treatment of LSD with a frequency of treatment of once per month. A high economic loss was incurred for supportive veterinary treatment of LSD during outbreak, estimated as 2286 ETB (US$99.42).

As a pastoralist said, 0.6% (3/74) of pregnant cows have aborted due to the diseases. Further, animals that recovered were no longer fit for export purposes and were therefore sold at local markets at a lower price. Lastly, the survey found that animals that had recovered from LSD produced less milk and suffered a loss in draught power (Figure 7).

## 4. Discussion

The present study reported an outbreak of lumpy skin disease that occurred at the end of November 2016. As far as the objective is to characterize LSDV from an outbreak cases, the occurrence of LSD was examined using clinical diagnosis, PCR, and virus isolation. Accordingly, out of 20 typical clinical cases sampled and tested in the present study, 10 were confirmed as positive for LSD using PCR.

The clinical manifestations observed in the present finding, such as fever, circumscribed nodules on the skin, necrotic nodules, enlargement of superficial lymph nodes, and lacrimation, are in agreement with those documented by [9, 16, 17] in different areas of the country. Host susceptibility, age, immunological status of the animal, dose, and route of virus inoculation affect the severity of disease [18].

In the present study, the observed morbidity rate (18%) is slightly higher than that reported in [9, 17], which recorded 6.1% and 13.61%, respectively, in different parts of the country. Other authors reported wide ranges of morbidity rates ranging from 3% up to 85% [6, 19]. Moreover, it is far higher than that reported in [20] that indicated that the usual morbidity rate is within a range of 1 up to 5%. In outbreaks of the disease, the morbidity rate varies depending on host susceptibility and the abundance of mechanical arthropod vectors.

With regard to mortality rate, the present finding report (1.34%) is slightly lower than that reported in [9] that reported 4.97%. The current finding agreed with the report of [17] that recorded 1.8% in feedlot. The authors in [21] also reported higher mortality rates above 5%. In the present study, the observed case fatality rate (7.44%) is also lower than that reported in [9, 17] that reported 36.48% and 30%, respectively, at different parts of the country. However, the latter was conducted on feedlot cattle and the result is based on clinical diagnosis, which may not be suitable for direct comparison.

In the present study, LSDV was isolated from samples collected from naturally infected cattle by inoculation on Vero cell. Characteristic pock lesions were observed after 1st passage and become clear after 3rd passage; this finding agrees with [22, 23] that successfully cultivated LSDV to detect the characteristic pock lesions.

The CPEs characterized by rounding of cells, aggregation of dead cells, and destruction of monolayers are in line with the reports made by [9]. PCR technique is highly suggested by different authors as a means to confirm of LSD from clinical specimens [9, 24].

In agreement with the present finding, the authors in [24] indicated that, on average, skin nodule samples exhibited higher concentration of virus than other samples, as evidenced by the lower average Ct values observed in PCR testing.

PCR was the test of choice for rapid detection and identification of the LSD outbreak causative agent. The PCR assay used in this work showed high specificity as a unique band of the expected size (172 bp) was obtained for DNA samples derived from skin biopsies, nasal swabs, and Neethling reference strain of LSDV.

The present study outbreak was reported at the end of November 2016, which is after the end of the main rainy season in most parts of the lowland and some highland agroecological zones. The seasonality of the outbreaks was also substantiated by questionnaire respondents who provided information on active disease surveillance. This agrees with the report of [9] that indicated that the disease is higher during rainy season and decreases in the dry season.

Other environmental risk factors associated with spread of LSD were found to be warm humid agroclimate, communal grazing/watering, and introduction of new animals in a herd. The incidence of LSD occurrence is high during wet seasons when biting-fly populations are abundant and it decreases or ceases during the dry season [16].

The findings associated with LSD mortality and veterinary expenses for treating sick animals suggest heavy losses in the sector. In line with current findings, various studies showed that lumpy skin disease causes severe economic losses as a result of the prolonged debilitating clinical course of the illness, reduced weight gain, temporary or permanent loss of milk production, infertility problems or even sterility in bulls, abortions in pregnant cows, and permanent damage to hides [9].

## 5. Conclusion and Recommendations

LSD was found to be the major cattle health problem that causes severe economic loss because of permanent damage to hides, a prolonged debilitating clinical course, reduced weight gain, temporary or permanent loss of milk production, temporary or permanent infertility or even sterility in bulls, and abortion of pregnant cows. In the present study, LSD causes significant effect on morbidity and mortality of animals that leads to economic loss because of abortion of pregnant cows, milk yield reduction, cost of dead animals, and cost of treatment. From the outbreak investigated in the present study, all age groups of animals were infected with significantly higher morbidity and mortality rate in young animals than other groups. Extracted DNA from skin biopsies and nasal swabs tested by using PCR revealed that all tested samples were LSDV. This implies failure of LSD vaccines, because a studied group of animals was annually vaccinated against the disease. Lumpy skin disease is considered as transboundary and trade band disease which has significant impediment on livestock market and animal products. Based on the above conclusions, the following recommendations are forwarded:

  Increasing appropriate handling facilities of vaccine besides providing quality vaccine and good administration skills should be the major considerations because vaccination is the only effective method to control the LSD in endemic countries like Ethiopia
  The government should establish strategic policies for effective control and eradication of the disease, i.e., strategic vaccination program and restriction of livestock movement
   Availability of a simple diagnostic test that can help confirm the cases at the field level is important to take control measures early during its occurrence
  More investigations should be carried out on the economic impact of LSD and the methods of spread, particularly the involvement of vectors
  Detailed molecular analysis of different isolates within the country needs to be carried out for further confirmation

## Figures and Tables

**Table 1:** Morbidity rate, mortality rate, and case fatality rates of LSD outbreaks in the study area.

Name of kebeles	No. of susceptible cattle	No. of affected cattle	No. of deaths	Morbidity rate (%)	Mortality rate (%)	Case fatality
Arda Galma	186	39	2	20.96	1.08	5.13
Kore Korme	336	55	5	16.36	1.49	9.09
Total	522	94	7	18.00	1.34	7.44

**Table 2:** LSD morbidity rate in different age and sex groups.

Risk factors	No. of cattle at risk	No. of cattle affected	Morbidity rate (%)	*X* ^2^	*P* value
Age					
<2	107	31	29.71	14.562	0.001
≥2–4	314	41	13.05		
>4	101	22	21.78		
Total	522	94	18.00		

Sex					
F	369	76	20.59	11.554	0.003
M	153	18	11.76		
Total	522	94	18.00		

**Table 3:** Mortality rate in association with risk factors of age and sex.

Risk factors	No. of cattle at risk	No. of cattle affected	No. of deaths	Mortality rate (%)	*X* ^2^	*P* value	Case fatality
Age							
<2	107	31	5	4.67	11.55	0.003	16.12
>2–4	314	41	1	0.31			7.14
>4	101	22	1	0.99			4.54
Total	522	94	7	1.34			7.44

Sex							
F	369	76	4	1.08	0.62	0.428	5.2
M	153	18	3	1.96			16.66
Total	522	94	7	1.34			7.44

## Data Availability

The data used to support the findings of this study are included within the article.
